# Enhanced ADCC Activity of a C-Terminal Lysine Variant of an IgG_1_ Antibody Driven by N-Linked MAN5 Glycan Using a Reporter Gene Assay

**DOI:** 10.3390/antib14040089

**Published:** 2025-10-17

**Authors:** Ming-Ching Hsieh, Kristiina Dorofejeva, Jingming Zhang, Diane L. Vy, Jun Qian, Alice M. Matathia, Timothy Blanc, Chao Richard Li, Babita S. Parekh

**Affiliations:** 1 Analytical Sciences, Eli Lilly and the Company, Branchburg, NJ 08876, USA; 2 Analytical Development, Eli Lilly and the Company, Indianapolis, IN 46221, USA; 3 Quality Control Compliance, Eli Lilly and the Company, Branchburg, NJ 08876, USA; 4 Manufacturing Process Development, Eli Lilly and Company, Branchburg, NJ 08876, USA; 5 Manufacturing Statistics, Eli Lilly and Company, Indianapolis, IN 46221, USA

**Keywords:** C-terminal lysine, MAN5 glycan, glycosylation, antibody-dependent cellular cytotoxicity (ADCC), reporter gene assay

## Abstract

Background: Antibody-dependent cellular cytotoxicity (ADCC) is an immune response where antibodies bind to target cells and activate effector cells through Fcγ receptors, ultimately leading to the destruction of the target cells. Methods: This study examined the ADCC activities of charge variants of a therapeutic IgG_1_, MAB1, using an internally developed reporter gene assay. In this assay, the proprietary target was expressed on DiFi cells, while FcγRIIIa was expressed on Jurkat effector cells. Results: The results revealed that different charge variants had varying levels of ADCC activity, with variants containing C-terminal lysine residues showing enhanced activity. The charge variants arose from modifications such as the presence of sialic acid at the glycan moiety, deamidation, and C-terminal lysine truncation, including K2 (two C-terminal lysine residues), K1 (one C-terminal lysine residue), and K0 (no C-terminal lysine residues) variants. Notably, the K1 and K2 variants demonstrated higher ADCC activity compared to the K0 and acidic variants. However, the observed increase was attributed not to the lysine residue itself, but rather to the MAN5 glycan associated with the lysine-containing variants. Conclusion: These findings challenge previous assumptions about the role of C-terminal lysine in ADCC, suggesting a shift in understanding the functional significance of charge variants and emphasizing the critical influence of glycan composition in therapeutic antibody efficacy.

## 1. Introduction

IgG_1_ antibodies are the most abundant subclass of immunoglobulin G in human circulation and are widely used in therapeutic applications due to their target specificity and the ability to mediate immune effector functions. One key mechanism of action, aside from specific target blockage, is antibody-dependent cellular cytotoxicity (ADCC). The process involves the variable region of the antibody (Fab) binding to the antigen on the target cell and the crystallizable region of the antibody (Fc) binding to the Fcγ receptors, particularly FcγRIIIa, on effector cells. Once activated, the effector cells release toxic substances, such as perforins and granzymes, to induce cell death [1].

Charge heterogeneity is a common characteristic of therapeutic monoclonal antibodies, often caused by post-translational modifications (PTMs) [2,3,4,5,6], such as deamidation, isomerization, glycation, sialylation, and C-terminal lysine variation, or by conformational changes [7] as a result of oxidation or other PTMs. Among the post-translational modifications, C-terminal lysine was often observed in therapeutic antibodies [8,9,10]. C-terminal lysine heterogeneity arises from incomplete carboxypeptidase-like activity during cell culture, resulting in three charge variants: K0, K1, and K2 [9,11,12]. Although C-terminal lysine is rapidly removed in vivo [13], studies suggest that its variation typically does not affect antigen binding [14], Fcγ receptor binding, neonate receptor (FcRn) binding, as well as complement-dependent cytotoxicity (CDC) activity [15,16,17]. The C-terminal lysine variants (K0, K1, and K2) of a human IgG_1_ antibody did not show differences in biophysical characteristics, nor in binding to human FcγRIIIa-Val^176^ by surface plasmon resonance (SPR) and to FcRn by affinity chromatography [18]. The conformations of C-terminal lysine variants of another IgG_1_ were found to be similar in terms of H/D exchange, solution dynamics, unfolding profile, and stability [19]. Moreover, in a study of a bevacizumab biosimilar product, the lysine variants exhibited similar structures, and the enriched lysine-containing variant did not improve the biological activity when 1:1 mixing with the main peak (K0) [20].

Although the C-terminal lysine does not appear to be crucial for biological functions, several studies have suggested that it may influence the potency of therapeutic antibodies. According to van den Bremer et al., the removal of the C-terminal lysine is essential for complement activation [21]. Their research found that the K2 form of the antibody was incompatible with the formation of ordered hexamers, which are critical for maximum complement-dependent cytotoxicity (CDC) at the cell surface. In contrast, the K1 variant was able to form hexamers and retain its CDC activity. Hintersteiner et al. [22] reported that the basic charge variants of a chimeric anti-GD2 antibody produced in Chinese hamster ovary (CHO) cells exhibited increased binding affinity to both Fc RγIIIa and neonatal receptor (FcRn). Subsequently, the same researchers examined other therapeutic antibodies and found that in three out of seven cases, the basic variants had a higher affinity to Fc RγIIIa and improved ADCC activities [23]. They attributed these altered biological properties to the surface charge of the protein.

Further investigation into the pharmacokinetics (PK) and efficacy of bevacizumab charge variants (K0, K1, and K2) was conducted in mice [24]. The K1 variant exhibited nearly four times higher FcRn binding affinity compared to K0, whereas the K2 variant showed about two times higher. Additionally, the K1 variant presented superior bioavailability relative to K0, whereas the K2 variant demonstrated lower bioavailability than K0, resulting in greater anti-tumor activity for the K1 variant [24].

Most therapeutic antibodies typically function by directly targeting their specific antigens, which can be either free ligands or membrane-bound receptors. Another prevalent mechanism of action for therapeutic antibodies, particularly IgG_1_ and IgG_2_, is antibody-dependent cellular cytotoxicity (ADCC). Two major factors that will impact ADCC activity include N-linked afucosylated glycans [25] and deamidation of N325–VSNK-containing peptide [21]. A wealth of evidence shows that the absence of fucose at the basal N-acetylglucosamine (GlcNAc) enhances ADCC activity by increasing Fc-FcγRIIIa receptor affinity, leading drug developers to adopt strategies for producing afucosylated antibodies for improved efficacy [26].

The present study reveals that the enhanced ADCC activity, as measured by an internally developed reporter gene assay [27], in C-terminal lysine charge variants of a therapeutic IgG_1_ antibody is driven by the MAN5 glycans associated with these variants, rather than the C-terminal lysine itself. The surrogate reporter gene ADCC assay employs target cells such as DiFi cells expressing the proprietary target on the surface and the effector cells, Jurkat cells engineered to stably express both the FcγRIIIa receptor and a luciferase reporter gene positioned downstream of the FcγRIIIa signaling pathway [28]. Although this approach does not directly measure cell death, as traditional ADCC assays using peripheral blood mononuclear cells (PBMCs) do, it provides superior consistency, accuracy, and throughput [29]. Comparative studies indicate that the ADCC reporter gene assay performs favorably relative to PBMC-based ADCC assays, particularly in terms of assessing alterations in antibody Fc-glycoform structures and different isotypes. Thus, it serves as an effective surrogate for determining ADCC activity. Additionally, the reporter gene assay’s demonstrated accuracy, precision, and robustness make it highly suitable for implementation in Quality Control laboratories for purposes such as drug release and stability testing [27].

The finding in this study challenges previous assumptions about the role of C-terminal lysine in ADCC, offering a paradigm shift in understanding the functional impact of charge variants and highlighting the critical role of glycan composition in therapeutic antibody activity.

## 2. Materials and Methods

### 2.1. Materials

#### 2.1.1. Cell Lines and Proteins

The cell lines used in the assay system are DiFi (Assay-Ready) cells (target cells) and Jurkat-vv-NFAT-Luc effector cells from Eli Lilly and Company (Indianapolis, IN, USA). The target receptor of MAB1 is expressed on these DiFi cells, while FcγIIIa is expressed on the Jurkat cells. The fully human monoclonal antibody, MAB1, and its native antigen were produced in NS0 and CHO cells, respectively, and manufactured at Eli Lilly and Company. Fc gamma receptor IIIa (FcγRIIIa)/CD16a–176Val and neonatal Fc receptor (FcRn) were purchased from R&D Systems (Minneapolis, MN, USA) and Sino Biological US Inc. (Wayne, PA, USA), respectively. Fc gamma receptor I (FcγRI)/CD16a was purchased either from R&D Systems or Sino Biological US. Peroxidase-conjugated AffiniPure Goat Anti-Human IgG, F(ab’)_2_ Fragment Specific was purchased from Jackson ImmunoResearch Laboratories, Inc. (West Grove, PA, USA).

#### 2.1.2. Reagents

The cell culture media used for effector cells (all from Gibco^TM^, ThermoFisher Scientific, Waltham, MA, USA, except otherwise indicated) are RPMI-1640 with L-glutamine and 25 mM HEPES supplemented with 10% heat-inactivated fetal bovine serum and L-glutamine, MEM-NEAA, sodium pyruvate, geneticin, hygromycin B, and puromycin dihydrochloride (Sigma-Aldrich, MilliporeSigma, Burlington, MA, USA). Hank’s Balanced Salt Solution (HBSS) without calcium and magnesium (Thermo Fisher Scientific, Waltham, MA, USA) is used to wash cells upon thaw.

Complete media for target cells includes 10% fetal bovine serum in DMEM/F12 with L-glutamine, 15 mM HEPES, and penicillin–streptomycin (pen/strep), all purchased from Gibco^TM^, Thermo Fisher Scientific (Waltham, MA, USA).

Serum-free assay media used for assay setup and dilutions includes RPMI-1640 with pen-strep-glutamine, MEM-NEAA, and sodium pyruvate, all purchased from Gibco^TM^, Thermo Fisher Scientific (Waltham, MA, USA). Bio-Glo^®^ Assay Reagent, Luciferase Assay System was purchased from Promega Corporation (Madison, WI, USA). Nonfat dry milk (Blotting Grade) was obtained from Bio-Rad (Hercules, CA, USA). Dulbecco’s phosphate-buffered saline was purchased from Gibco^TM^ (ThermoFisher Scientific). TMB (3,3′5,5′-tetramethylbenzidine) peroxidase substrate (2 components) was obtained from Kirkegaard & Perry Laboratories (KPL) Inc. (Gaithersburg, MD, USA).

#### 2.1.3. Other Chemicals

Trifluoroacetic acid (TFA, sequence grade) and acetonitrile were purchased from Thermo Fisher Scientific Pierce^TM^ (Rockford, IL, USA) and from Mallinckrodt Baker, Inc (J.T. Baker, Phillipsburg, NJ, USA), respectively. Trypsin (sequencing grade TPCK modified) and CellTiter-Glo^®^ were obtained from Promega Corporation (Madison, MI, USA). Carboxypeptidase B (CPB) was obtained from Roche Diagnostics (Indianapolis, IN, USA). Guanidine hydrochloride (GudHCl), Tris base, methanol, sodium phosphate salts, acetic acid, sodium chloride, Tween 20^®^, sulfuric acid, 6 N HCl, and 10 N sodium hydroxide were purchased from Mallinckrodt Baker, Inc. (J.T. Baker, Phillipsburg, NJ, USA). Dithiothreitol (DTT), iodoacetamide, anthranilic acid, 2, 5-dihydroxybenzoic acid (DHB), Coomassie Blue, and 2-picoline borane complex were purchased from Sigma-Aldrich (MilliporeSigma, Burlington, MA, USA). For HPLC buffers and solvents, 18 MΩ water from Millipore Milli-Q^®^ Integral 5 Water Purification System, MilliporeSigma (Burlington, MA, USA) was used.

### 2.2. Ion Exchange Chromatography (IEC)

Protein samples were desalted using a Sephadex G-25 cartridge (NAP-5, Cytiva, Marlborough, MA, USA), following the manufacturer’s instructions prior to ion exchange chromatography. Ion exchange chromatography was conducted with an analytical weak cation exchange column (ProPac WCX-10, 250 × 4.0 mm, 10 µ, Thermo Fisher Scientific) using an Agilent 1100 series HPLC (Agilent Technologies, Wilmington, DE, USA). Protein (total of 50 µg) was monitored at 214 and 280 nm. Buffer A was 10 mM sodium phosphate, pH 7.2 ± 0.1, and Buffer B was 200 mM NaCl in 10 mM sodium phosphate buffer, pH 7.2 ± 0.1. The gradient started from 100%A to 22.5%B in 90 min at a flow rate of 0.5 mL/min. The column temperature was kept at 50 °C and was equilibrated with Buffer A for 15 min prior to the next injection.

MAB1 charge isoforms were collected using an analytical Agilent HPLC, equipped with a 1260 Infinity bio-inert analytical-scale fraction collector. Fraction collection was based on the UV trace at specific time slices and temperature was set at 4 °C. Solution in each fractions was concentrated using an Amicon^®^ Ultra-4 Centrifugal Filter Unit (MilliporeSigam, Burlington, MA, USA) at 4 °C with a benchtop centrifuge (Sorvall^TM^ ST 40R, Thermo Scientific, Waltham, MA, USA). A four-fold formulation buffer (10 mM Sodium Citrate, 133 mM Glycine, 50 mM Mannitol, and 40 mM Sodium Chloride at pH 6.0) was added to the sample at a 1:3 ratio in an Amicon^®^ Ultra-4 centrifugal filter unit to prevent MAB1 from fragmentation during centrifugation. Collected charge isoforms were frozen at −20 °C upon further analyses.

### 2.3. ADCC Cell-Based Reporter Gene Assay

Target cells were thawed, washed, and seeded at 20,000 cells per well in 96-well white tissue culture plates for overnight incubation. The next day, MAB1 was serially diluted in a dose–response manner and added to the assay plates along with effector cells at 100,000 cells per well concentration. The mixture was incubated for approximately 4 h at 37 °C with 5% CO_2_. Promega’s Bio-Glo was then added to measure luciferase production on a luminometer.

ADCC activity of MAB1 was determined by comparison of the sample to the assigned independent reference standard (unfractionated MAB1). Full 8-point dose–response curves were plotted and assessed using the 4-parameter nonlinear logistic regression model (4-PL curve). Half-maximal effective concentration (EC_50_) for the test sample and reference standard (ratio) was calculated after the assay had met all acceptance criteria for system suitability. The ADCC activity is defined as the ratio of EC_50_ of the test sample to the EC_50_ of the reference standard in terms of percentage.

### 2.4. IEF (Isoelectric Focusing) by Agarose Gel Electrophoresis and Western Blot

IEF gel electrophoresis was performed using the Multiphor II Electrophoresis System (GE Healthcare Life Sciences, Piscataway, NJ, USA). IEC fraction samples were desalted using Pierce Zeba desalting columns (ThermoFisher Scientific, Waltham, MA, USA) with deionized water. The unfractionated sample was adjusted to 1 mg/mL and all other fractions were adjusted to 0.23 mg/mL. An aliquot of 10 µL from each desalted sample (10 µg and 2.3 µg of unfractionated and fractions, respectively) was applied to the Precast IsoGel™ Agarose IEF gel with a pH gradient of 6.5 to 10 (Lonza, Wakersville, MD, USA). The gel was pre-focused for 20 min at 1000 V, maximum mA, and 1 W, then focused for 90 min at 1000 V, maximum mA, and 25 W. The temperature was kept at 10 °C. The IEF makers were the High pI Kit pH 5.0–10.5 from Cytiva (Marlborough, MA, USA) and protein samples were visualized by staining with Coomassie Blue.

The IEF-separated IEC fractions were transferred to a PVDF membrane (Cytiva) by press blotting for 5 min. The PVDF membrane was first rehydrated with 100% methanol and then 0.2 M Tris-HCl (pH 8). The equilibrated PVDF membrane was placed on the IEF gel, and proteins were transferred to the PVDF membrane by contact. After transfer, the membrane was blocked with KPL milk blocker (SeraCare, Milford, MA, USA) for 1 h, followed by overnight incubation with the ligand conjugated with horse radish peroxidase. The blot was washed three times with KPL milk washing buffer and developed with TMB substrate for 5–10 min.

### 2.5. Cell-Based Potency Assay

The cell-based potency assay was used to evaluate the ability of the MAB1 antibody to inhibit the proliferation of DiFi cells, which are human colon adenocarcinoma cells.

In this assay, DiFi cells (1 × 10^4^ cells/well) were seeded into a 96-well plate (NUNCTM white flat bottom plate, ThermoFisher Scientific, Waltham, MA, USA). Serial dilutions of MAB1 were prepared and added at concentrations ranging from 5 nM to 0.13 nM. The cells were then incubated for 96 h at 37 °C with 7% CO_2_. After the incubation period, CellTiter-Glo^®^ substrate (Promega, Madison, WI, USA) was added to the wells, and the plate was shaken on an orbital shaker at room temperature for 60 min. Finally, luminescence was measured using a SpectraMax L plate reader (Molecular Devices, San Jose, CA, USA). The relative potency of the samples was then calculated as the ratio of the IC50 value of the MAB1 reference standard to that of the samples.

Cell viability is determined based on the amount of ATP generated by the metabolically active cells in the culture. ATP production is measured in this assay by converting luciferin to oxyluciferin in the presence of ATP. The light emitted during this process is detected using a plate reader equipped for luminescence measurement. The intensity of the light signal is proportional to the amount of ATP present, which in turn correlates with the number of viable cells. Higher luminescence indicates a greater number of viable cells and less inhibition of DiFi cell growth.

### 2.6. Biacore Binding Assay

Biacore binding assay is a label-free technique for measuring the interactions between two molecules through surface plasmon resonance (SPR) in terms of Resonance Units (RUs), using a Biacore^TM^ T100/T200 system (Cytiva, Marlborough, MA, USA). Probe molecules, e.g., the native ligand of MAB1, neonatal Fc receptor (FcRn), and Fc gamma receptor IIIa (FcγRIIIa or CD16a), were immobilized on a CM5 chip (Cytiva) at a specific concentration. Serially diluted MAB1 samples were then injected into the CM5 sensor chip. With the MAB1 reference standard, a standard calibration curve was created with different concentration ranges, according to the specific probe molecules (described in the subsections). The calculated concentrations of the samples were based on a 4-parameter regression analysis of the standard curve. The binding activity, reported as % activity, was calculated from the median calculated concentration of MAB1 from five dilutions divided by the protein concentration (determined by A280) × 100.

#### 2.6.1. MAB1 Native Antigen Binding Assay

The MAB1 native antigen was immobilized on a CM5 chip at 50 µg/mL in 10 mM sodium acetate buffer, pH 4. With the MAB1 reference standard, a standard calibration curve was created with concentrations from 2000 ng/mL to 78 ng/mL. The samples were tested at five concentrations from ~890 ng/mL to ~120 ng/mL, based on the nominal protein concentration determined by A280. The chip can be regenerated by washing with 5 M NaCl in 50 mM NaOH solution.

#### 2.6.2. FcRn Binding Assay

FcRn at 10 µg/mL was immobilized on a CM5 chip in 10 mM sodium acetate buffer pH 4.5. Because human IgG binds to FcRn with high affinity at acidic pH and dissociates at neutral pH, the binding is performed at an acidic pH of 5.8 and the chip is regenerated at a neutral pH of 7.2. The standard calibration curve was generated using the MAB1 reference standard with the concentration ranges from 3000 ng/mL to 117 ng/mL. The samples were tested at five different concentrations ranging from 198 ng/mL to 1000 ng/mL based on the nominal protein concentration determined by A280.

#### 2.6.3. CD16a Binding Assay

The CD16a protein was immobilized on a CM5 chip at 50 µg/mL in 10 mM sodium acetate buffer, pH 5.5. A calibration curve is created with the reference standard curve spanning from 17.95 to 460 µg/mL. The samples are tested at five different concentrations from 202.5 µg/mL to 40 µg/mL based on the nominal protein concentration determined by A280.

### 2.7. CD64 ELISA Binding Assay

The CD64 protein at a concentration of 3 µg/mL in PBS was coated on a 96-well plate (Nunc Immuno^TM^ MaxiSorp^TM^ Flat Well Plate, ThermoFisher Scientific, Waltham, MA, USA). We aspirated the wells and washed them five times with 1X PBS containing 0.05% Tween 20. Nonfat dry milk (5%) was added to each well to block non-specific sites in the wells. After washing and blotting dry, serial dilutions of the MAB1 reference standard and samples were added to the wells. Protein concentrations were from 9 µg/mL to 0.15 ng/mL for the reference standard and samples. The plate was allowed to stand at room temperature for 2 h. After washing and blotting dry again, the goat anti-human IgG, F(ab’)_2_ conjugated with peroxidase in 5% nonfat dry milk was added. The plate was sealed and incubated at room temperature for an hour. The TMB substrate solution was added after the plate was washed and blotted dry and allowed to incubate at room temperature for 15 min before adding 4 N sulfuric acid. The plate was read by a plate reader (SpectraMax 5, Molecular Devices, San Jose, CA, USA) at an absorbance of 450 nm.

### 2.8. Physical Characterization of MAB1 and Charged Isoforms

#### 2.8.1. Thermal Stability with Differential Scanning Calorimetry (DSC)

Differential scanning calorimetry was performed with Microcal^TM^ VP-DSC (Malvern Panalytical, Northhampton, MA, USA). Thermal ramps were performed from 10 to 110 °C with scan rates of 200 °C/h with a pre-scan time of 3 min and a filtering period of 16 s. The protein concentration of ~2.8 µM was used.

#### 2.8.2. Intrinsic Fluorescence

Equilibrium tryptophan fluorescence emission spectra were collected on a HORIBA Jobin Yvon FluoroLog-3 fluorometer (Horiba Scientific, Piscataway, NJ, USA) equipped with double-grating monochromators in both excitation and emission positions for better stray-light rejection. The data were recorded with a 1 cm quartz cuvette, 3 nm excitation and 2 nm emission bandwidths, and 0.3 s integration for each wavelength. These spectra were collected at 25 °C with 295 nm excitation from 305 to 450 nm with 0.5 nm spacing. Protein concentration was 0.05 mg/mL in 10 mM sodium phosphate with 15% NaCl. A280 was measured and used to normalize the tryptophan fluorescence spectrum.

### 2.9. Carboxypeptidase B (CPB) Treatment

Carboxypeptidase B was reconstituted with MilliQ water to make a 5 mg/mL stock. The enzyme stock was aliquoted and stored at −20 °C in a freezer upon use. The enzyme-to-substate ratio is 1:25 (*w*/*w*), and incubation was allowed at 37 °C for 3 h. The treated sample was directly subjected to IEC analysis.

### 2.10. Tryptic Peptide Mapping

Monoclonal antibody MAB1 samples (~20 µg) were brought to complete dryness under vacuum. The proteins were denatured and reduced by guanidine hydrochloride and DTT in 50 mM Tris–HCl at 50 °C for 30 min. After denaturation and reduction, the proteins were alkylated with iodoacetamide for 30 min in darkness. The reaction mixture was then diluted with 50 mM Tris–HCl pH 7.5. The trypsin digestion was performed at 37 °C for 5 h using an enzyme–protein ratio of 1:5 (*w*/*w*). The reaction was terminated by adding 20% TFA. The resultant peptides were resolved in a C18 reverse-phase column (Aeris widepore XB-C18, 200 Å, 3.6 µ, 250 × 2.1 mm, Phenomenex, Torrance, CA, USA) using an Agilent HPLC 1260 series (Agilent Technologies, Santa Clara, CA, USA) interfaced with an Exploris 480 mass spectrometry (ThermoFisher Scientific, San Jose, CA, USA) equipped with electrospray source. The temperature of the ion transfer tube was set at 320 °C. The spray voltage was set at 3.6 kV. The digested sample was eluted with a gradient from 0% solvent B to 40% solvent B (solvent A is 100% H_2_O with 0.05% TFA, solvent B is 100% acetonitrile with 0.04% TFA) in 50 min at a flow rate of 0.2 mL/min.

### 2.11. N-Linked Oligosaccharide Profiling with HILIC Analysis

Approximately 80 µg of MAB1 was brought to dryness by a SpeedVac (Thermo Scientific™ Savant™ SPD131DDA, Waltham, MA, USA). An aliquot of 50 µL of 1 X reaction buffer was added to dissolve the sample, then 3 µL of denaturation solution was added, followed by denaturation at 100 °C for 5 min. N-linked oligosaccharides of MAB1 were released by PNGase F (Agilent Technologies, Santa Clara, CA, USA) in the reaction buffer for 18 h at 37 °C. The release N-linked oligosaccharides were derivatized by anthranilic acid (2-AA) with 2-picoline borane complex at 70 °C for 90 min. The derivatized glycans were purified by solid-phase extraction (SPE) cartridge (Oasis^®^ HLB 1 cc from Waters, Milford, MA, USA). The N-linked oligosaccharides were eluted by 0.5 mL of 20% acetonitrile and stored at −20 °C freezer.

The purified 2-AA-labeled glycans were analyzed by UHPLC (1290 series, Agilent Technologies, Stata Clara, CA, USA) using an Acquity BEH Glycan column (1.7 µm 2.1 × 150 mm, Waters Corporation, Milford, MA, USA) with 100 mM ammonium formate as mobile phase A and acetonitrile as mobile phase B. The gradient started at 22.5% B and then went to 45% B in 40 min. Column temperature was kept at 60 °C with a flow rate of 0.2 mL/min.

### 2.12. QTOF-MALDI Mass Spectrometry Analysis of Anthranilic Acid-Labeled N-Glycans

Small aliquots of the reductively aminated N-glycans from IEC fractions were dialyzed using a floating disc of MF-Millipore^TM^ membrane filter (0.025 µm pore, 47 mm diameter) from MilliporeSigma (Burlington, MA, USA) on water for 45 min. The dialyzed samples were dried using a SpeedVac and redissolved in a small amount of water. The samples were then mixed with 2, 5-dihydroxybenzoic acid (DHB, 10 mg/mL) matrix in water/acetonitrile (70:30). The mixture was dried onto the MALDI target and analyzed using a QSTAR^®^ XL MALDI-TOF mass spectrometer (Applied Biosystems, AB Sciex, Framingham, MA, USA), operating in the negative-ion mode [30].

## 3. Results

### 3.1. Charge Variants of MAB1

**Figure 1:**
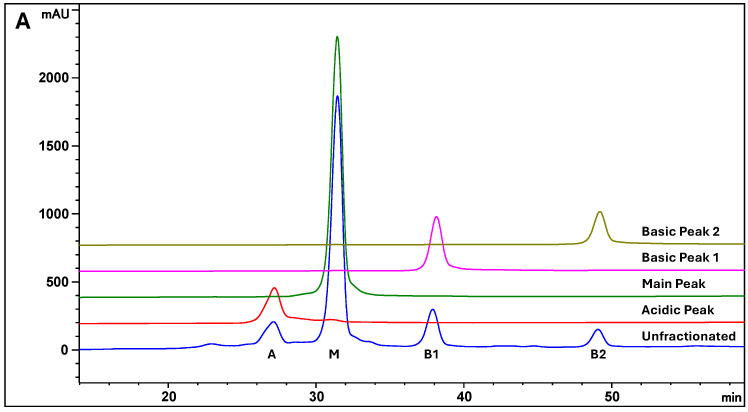

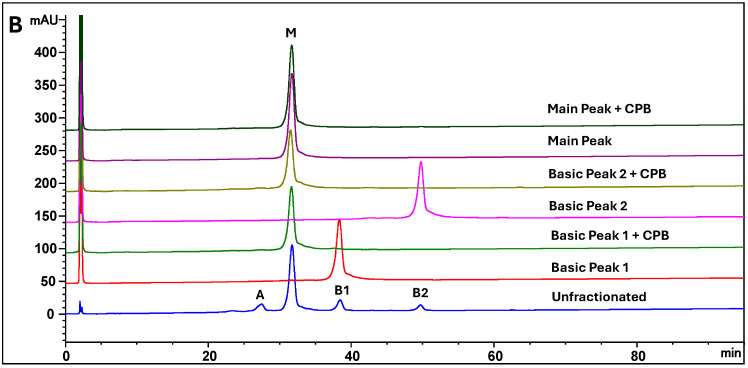
Charge Heterogeneity of MAB1 by Ion Exchange Chromatography. (**A**) Purity of the Isolated Peaks. (**B**) Carboxypeptidase B (CPB) removes the C-terminal lysine and moves the peaks B1 and B2 to the main peak position.

The ion exchange chromatography (IEC) profile of the therapeutic antibody MAB1, produced from a murine cell line (NS0), showed a typical charge heterogeneity profile with four major peaks (Figure 1A Unfractioned). The peaks were successfully isolated, resulting in an acidic peak (A), a main peak (M), and two basic peaks (B1 and B2), each exhibiting high purity (Figure 1A). The two basic peaks are C-terminal lysine-containing peaks. Treatment with Carboxypeptidase B (CPB) results in the removal of the C-terminal lysine, causing the basic peaks to shift to the main peak position (Figure 1B), which lacks sialic acid and C-terminal lysine. As a result, these peaks are identified as K1 and K2 variants, respectively. Furthermore, peptide mapping using LC-MS/MS confirmed the identities of B1 and B2. On the other hand, acidic peak A contains sialic acid (N-glycolylneuraminic acid or NGNA) in its glycan moiety, as indicated by the detection of at least two NGNA-containing glycans in hydrophilic interaction chromatography and four NGNA-containing glycans in the peptide mapping and MALDI mass spectra of acidic peak A, which are described in Section 3.3, Section 3.4 and Section 3.5.

### 3.2. Antibody-Dependent Cellular Cytotoxicity (ADCC) Activity of MAB1

**Figure 2:**
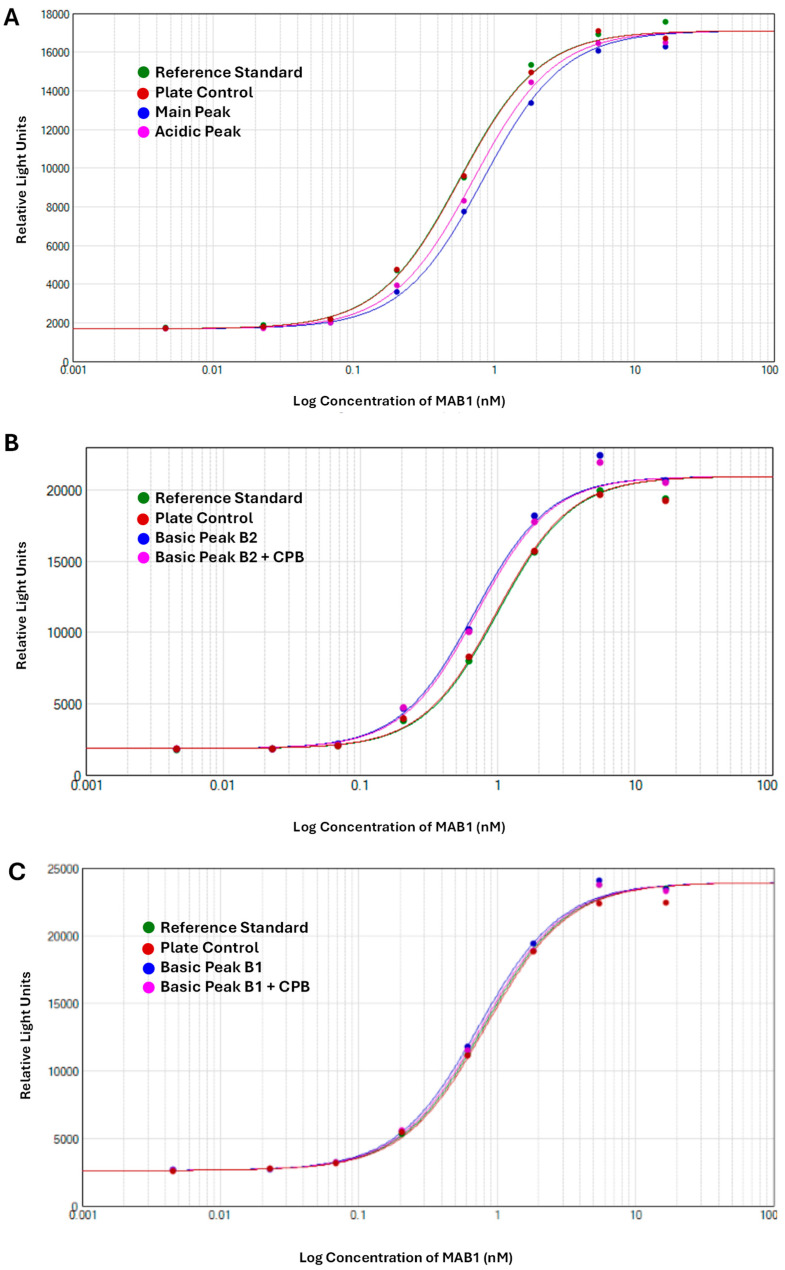
Examples of The Antibody-Dependent Cellular Cytotoxicity (ADCC) Activities of MAB1 IEC-Isolated Peaks Using Reporter Gene Assay. All data were fitted using the Four-Parameter Logistic (4PL) model. Panels illustrate (**A**) the fitted curves for the reference standard, plate control and 2 samples of Main Peak and Acidic Peak; (**B**) 2 samples of Basic Peak 2 (B1) and Basic Peak 2 (B2) treated with CPB; and (**C**) 2 samples of Basic Peak 1 (B1) and Basic Peak 1 (B1) treated with CPB.

**Figure 3:**
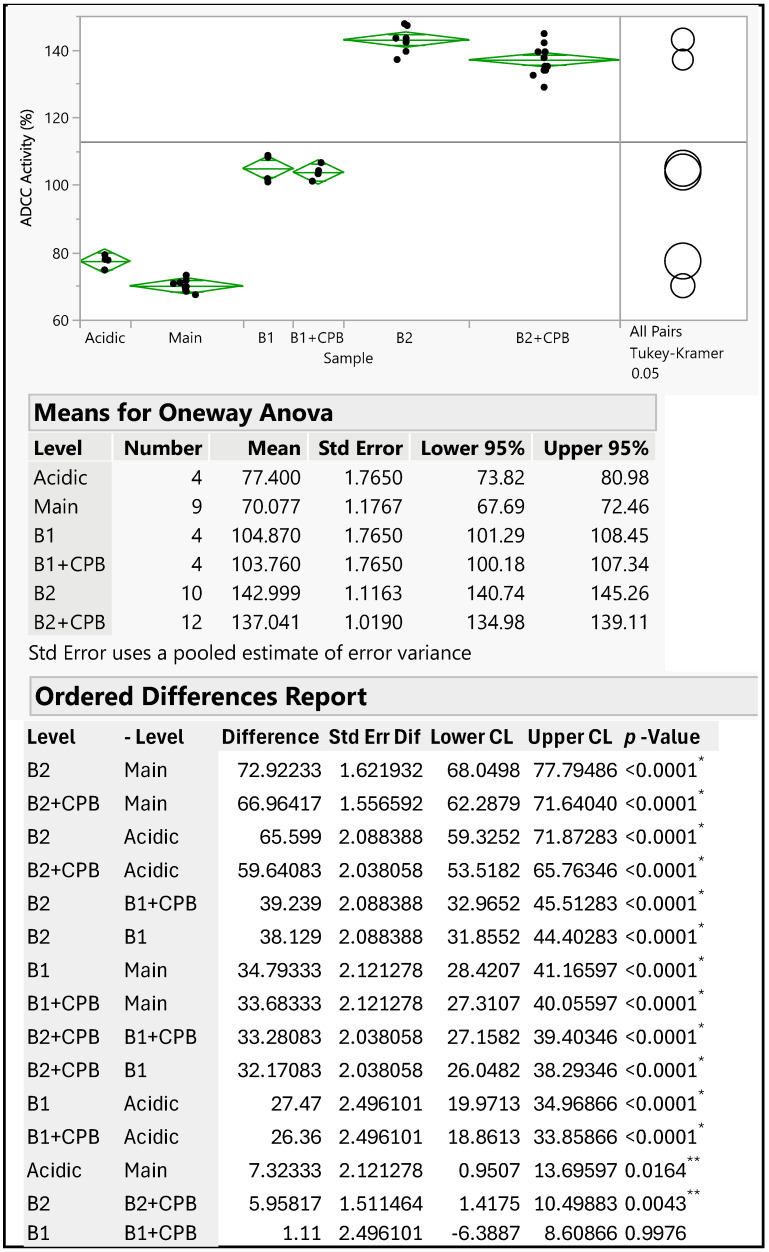
ANOVA Results of ADCC Activity of MAB1 IEC-Isolated Peaks (Software JMP^®^, Version 16.1.0). The “number” column indicates the number of plates used for triplicate analysis of the test articles. The Main Peak was evaluated in five independent setups across nine plates. Acidic Peak, B1, and B1 + CPB samples were each analyzed in two independent setups using a total of four plates. B2 and B2 + CPB samples underwent assessment in five independent setups across a total of twelve plates. *: indicating statistical significance; **: indicating statistically marginal significance.

The therapeutic antibody MAB1, an IgG_1_ subclass, was assessed for ADCC activity to monitor its effector function. The ADCC activities of IEC-isolated peaks were compared with the unfractionated MAB1 (reference standard) using the methods described in Section 2.3. The data were fitted into a sigmoidal curve with the Four-Parameter Logistic (4PL) model, as shown in Figure 2 as an example. This ADCC reporter gene assay was fully validated, demonstrating intermediate precision with a coefficient of variation (CV) of approximately 3%. Each result was obtained from the triplicate dilution of each test article, conducted alongside the reference standard (unfractionated MAB1) and plate control, which were also performed in triplicate. The “number” column in Figure 3 indicates the number of plates that were set up on the same or different days independently alongside mean values, standard deviations, and 95% confidence intervals. Results indicated that the average ADCC activities for Acidic Peak (A), Main Peak (M), Basic Peak 1 (B1), and Basic Peak 2 (B2) were 77%, 70%, 105%, and 143%, respectively (Table 1), where the unfractionated reference standard was 100%. An Analysis of Variance (ANOVA) using the Tukey–Kramer HSD method (JMP^®^, Version 16.1.0) showed that Basic Peak 1 (B1) and Basic Peak 2 (B2) had statistically higher ADCC activities than the Main Peak (*p*-value < 0.001, Figure 3). The ADCC activities of B1 and B2 were significantly different from the Main Peak, with mean differences of approximately 35% and 70%, respectively. The lower ADCC activities of Main Peak and Acidic Peak were reasonable, as the higher activity level of Basic Peak 1 and Basic Peak 2 contributed to an overall activity of 100% for unfractionated MAB1.

Interestingly, the enhanced ADCC activity was not linked to the C-terminal lysine, as its removal with CPB did not reduce the ADCC activity to the basal level observed in the Main Peak. The differences observed between B2 and CPB-treated B2 were minor (less than 6%), though marginally significant, likely due to assay variability. Nevertheless, both groups still showed significant differences compared to the Main Peak (Figure 3).

### 3.3. Glycosylation of MAB1 and the Charge Variants Analyzed by Hydrophilic Interaction Chromatography (HILIC)

**Figure 4:**
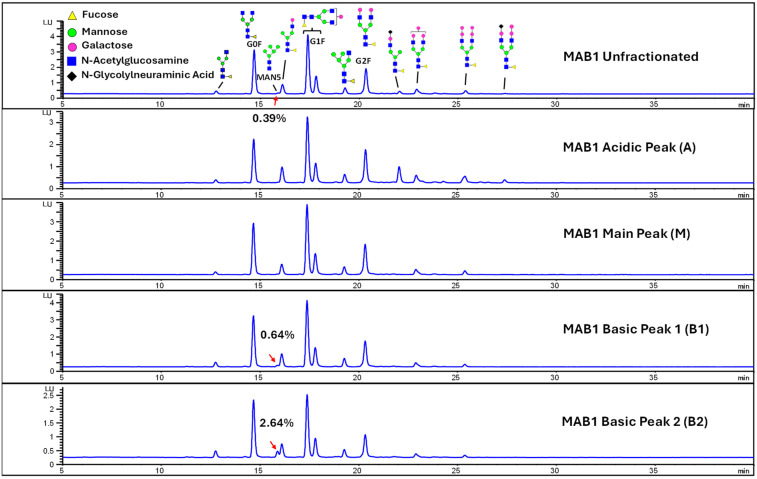
The HILIC Chromatographic Profiles of MAB1 Unfractionated and IEC-Isolated Peaks.

Considering that the C-terminal lysine did not account for the increased ADCC activity of Basic Peak 2, this prompted further investigation into potential factors contributing to this elevated activity. To explore this in more detail, the N-linked glycans of MAB1 were released using PNGase F and derivatized with anthranilic acid (2-aminobenzoic acid, 2-AA) via reductive animation [31]. The derivatized glycans were enriched using an Oasis^®^ HLB cartridge (Waters Corporation) and analyzed with an HILIC (Acquity BEH Glycan) column. The glycan structures corresponding to each peak are shown in Figure 4, alongside the HILIC chromatographic profiles. The MAN5 glycan (highlighted by the red arrow) in Basic Peak 2 (B2, ~2.6%) is more prominent compared to Basic Peak 1 (B1, ~0.6%) and unfractionated MAB1 (~0.4%) (Table 2). Additionally, sialic acid-containing glycans were predominantly found in the Acidic Peak (A) as expected. Other afucosylated glycans (such as G0, and G0 minus GlcNAc) are present in a minimal amount (0.1–0.2%) across the MAB1 samples (Table 2). Clearly, the MAN5 glycan was the major contributor to the afucosylation observed in Basic Peak 2 (B2).

### 3.4. Glycosylation of MAB1 and the Charge Variants Analyzed by QTOF-MALDI Mass Spectrometry

**Figure 5:**
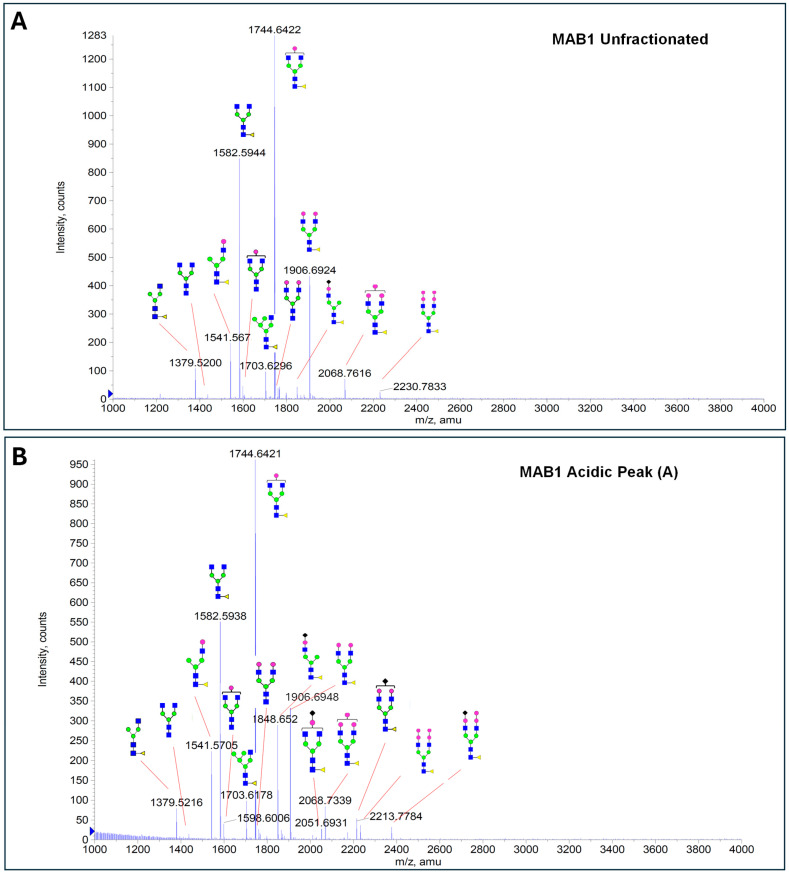

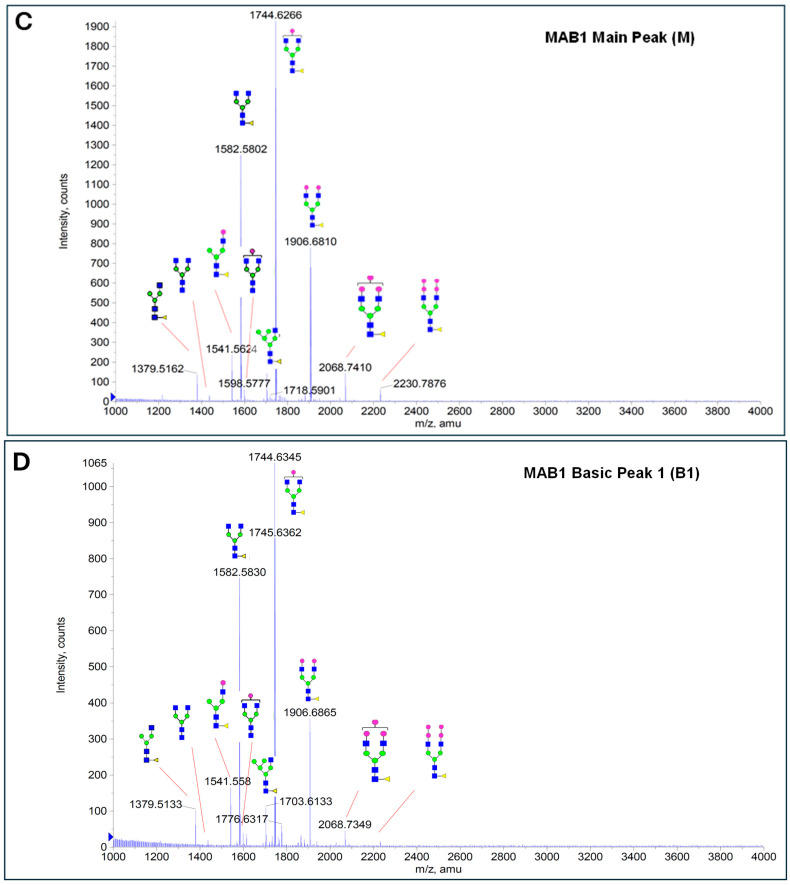

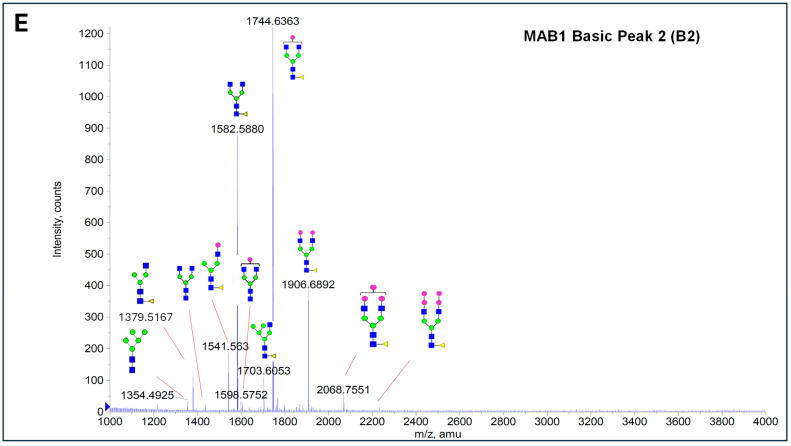
Mass Spectra of 2-AA-Labeled Glycans from Unfractionated MAB1 and IEC-Isolated Peaks. (**A**) Unfractionated MAB1; (**B**) Acidic Peak (A); (C) Main Peak (M); (**D**) Basic Peak 1 (B1); (**E**) Basic Peak 2 (B2). Monosaccharide residues are N-acetylglucosamine (GlcNAc, 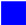), fucose (Fuc, 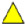), mannose (Man, 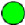), galactose (Gal, 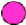), and N-glycolylneuraminic acid (NGNA, 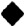).

The glycosylation of MAB1 and its charge variants isolated from IEC were further analyzed using a QSTAR mass spectrometer with a MALDI source. The 2-AA-labeled glycan structures found in MAB1 and their theoretical masses are listed in Table 3. The m/z spectra of 2-AA-labeled glycans from unfractionated MAB1 and the IEC-isolated peaks are shown in Figure 5. This method serves as a qualitatively orthogonal approach to HILIC analysis, with results that are well aligned in terms of glycan species. The MAN5 glycan was clearly observed in Basic Peak 2 (B2), confirming its association with this peak, as seen in the HILIC analysis (Figure 5). However, the MAN5 glycan was not detected in Basic Peak 1 (B1) in the mass spectrometry analysis, likely due to differences in sample preparation and the sensitivity of the method.

### 3.5. Peptide Mapping Analysis

Tryptic peptide mapping was performed to analyze glycopeptides and other post-translational modifications, including the deamidation of the VSNK peptide, which was found to be inversely correlated with ADCC activity [21]. The tryptic peptides were separated using reversed-phase HPLC, and their identities were confirmed by MS and MS/MS spectra. The quantities of different glycopeptides from unfractionated MAB1 and IEC-isolated peaks are presented in Table 4. Consistent with the HILIC data, the MAN5 glycan exhibited significantly higher abundance in the B2 peak, while the sialic acid (NGNA)-containing glycans were predominantly identified in the Acidic Peak (A). The MAN5 glycan in the B1 peak was slightly above the baseline. These results correlated with the findings from both HILIC and mass spectrometric analyses. Due to the high sensitivity of the mass spectrometer, MAN5 was detected at basal levels in both the Main Peak (M) and the Basic Peak 1 (B1).

In addition to glycopeptides, the peptide containing sequence VSN^329^K was also assessed for deamidation. Across all samples from both unfractionated MAB1 and IEC-isolated peaks, deamidation at N^329^ was minimal, ranging between 0 and 0.5% (Table 5). Therefore, the deamidation of the VSNK-containing peptide does not appear to impact the ADCC activity of MAB1 charge variants.

The three orthogonal approaches used to assess MAB1 glycan levels consistently demonstrated that the MAN5 glycan in Basic Peak 2 (B2) was present in higher quantities than any other peaks isolated via IEC. No other glycans from MAB1 significantly influenced ADCC activity. Subsequently, the physical and biological characteristics of these IEC-isolated peaks were evaluated to ascertain if the abundance of MAN5 glycan led to additional changes in protein structure, stability, binding to its native antigen, or other effector functions.

### 3.6. Intrinsic Fluorescence

**Figure 6:**
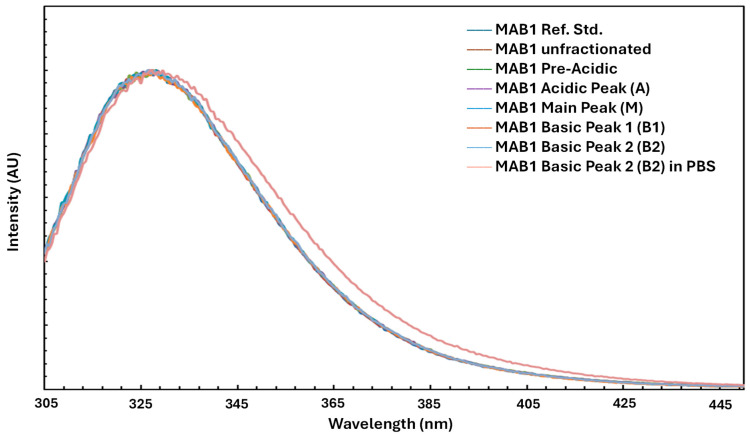
Intrinsic Fluorescent Spectra of Unfractionated MAB1 and IEC-isolated Peaks.

Tryptophan fluorescence, which is sensitive to changes in protein structure, was measured to assess secondary and tertiary modifications in MAB1 and IEC-isolated peaks using a FluoroLog-3 fluorometer (HORIBA Jobin Yvon, Piscataway, NJ, USA). Figure 6 shows the superimposed intrinsic fluorescence spectra of unfractionated MAB1 and IEC-isolated peaks, with their emission maxima summarized in Table 6. These results indicated no significant differences in tertiary structure among charge variants based on tryptophan fluorescence. However, Basic Peak 2 (B2) in PBS (compared to the formulation buffer) exhibited a higher λ_max_ (redshift) and broader width, suggesting that MAB1 B2 exists in a more hydrophilic environment (PBS) than in the formulation buffer.

### 3.7. Temperature-Induced Unfolding Study by Differential Scanning Calorimetry (DSC)

**Figure 7:**
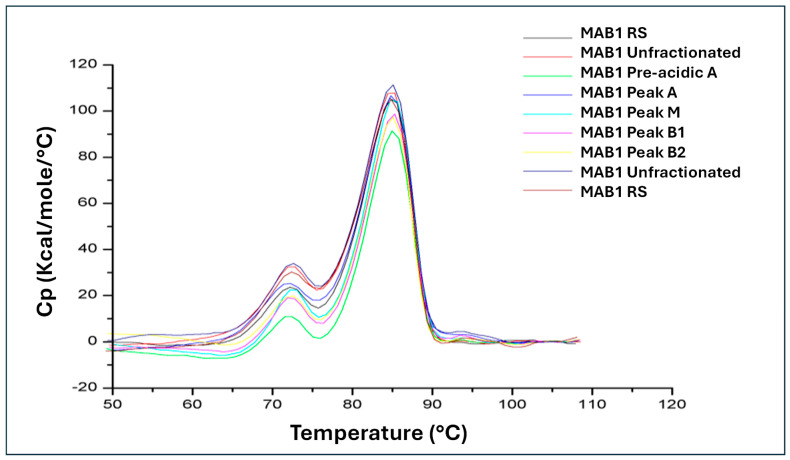
Temperature─Induced Thermograms of MAB1 and IEC-Isolated Peaks.

The unfolding temperature (Tm) of a protein is a critical thermodynamic property that reflects protein stability. The thermal stability for MAB1 and the IEC-isolated charge variants was measured using a VP-Capillary DSC Platform (Malvern Pananlytical). The thermograms of MAB1 and the IEC-isolated peaks are shown in Figure 7, depicting the typical two transitions for IgG_1_ unfolding [32]. Table 7 lists the melt temperatures, Tm_1_ (approximately 72 °C) associated with Fab unfolding and Tm_2_ (approximately 85 °C) associated with Fc unfolding, for all samples. The data revealed no differences in thermal stability among unfractionated MAB1 and the IEC-isolated peaks.

### 3.8. Western Blot Analysis of the Biological Binding Activity of MAB1 and IEC-Isolated Peaks

**Figure 8:**
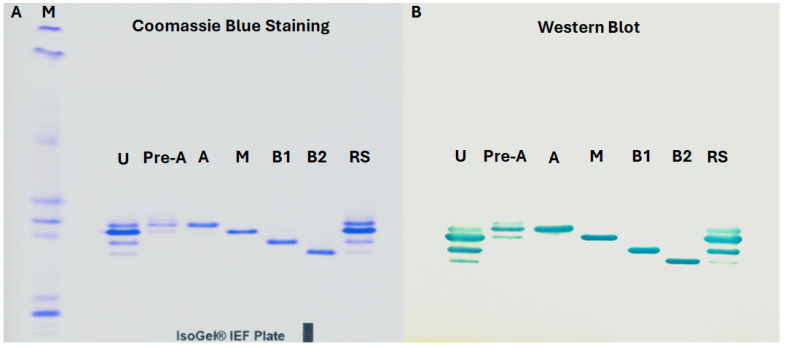
Isoelectric focusing (IEF) Gel Electrophoresis (**A**) and Western Blot Analysis (**B**) of Unfractionated MAB1 and IEC-Isolated Peaks. M: pI markers; U: unfractionated MAB1; Pre-A: Pre-Acidic Peak; A: Acidic Peak; M: Main Peak; B1: Basic Peak 1; B2: Basic Peak 2; RS: Reference Standard.

MAB1 charge variants were separated using isoelectric focusing (IEF) gel electrophoresis (Figure 8A) and transferred to a PVDF membrane. The MAB1 and its IEC-isolated variants were then hybridized with the native antigen conjugated with horseradish peroxidase. Upon addition of the TMB substrate, distinct bands corresponding to the target proteins became visible, as shown in Figure 8B. Western blot analysis confirmed that both unfractionated MAB1 and IEC-isolated peaks were able to bind the native antigen.

### 3.9. Cell-Based Proliferation Inhibition Assay for Unfractionated MAB1 and IEC-Isolated Peaks

A cell-based assay was used to further assess the biological activity by measuring cell growth inhibition in a dose-dependent manner. The 50% inhibitory concentration (IC_50_) was calculated and compared to the reference standard tested alongside the samples. Table 8 summarizes the percent biological activity for both the unfractionated MAB1 and the peaks isolated via IEC. Given the inherent natural variability of the assay, the acceptance range was defined as 70% and 130%. Consequently, there was no significant difference in the percent biological activity between these samples.

### 3.10. Biacore Binding Assays for Native Antigen, FcRn, and CD16a

Surface plasmon resonance (SPR) enables the examination of functional binding interactions between molecules without requiring labels. Biacore SPR biosensors were used for active concentration measurement and reported as percent activity against the standard. On the CM5 chip, three receptors were immobilized: the native antigen of MAB1, neonatal Fc receptor (FcRn), and Fc gamma receptor IIIa (FcγRIIIa, or CD16a). The percent activities of the unfractionated MAB1 and fractionated MAB peaks percent activities compared to the reference standard are detailed in Table 9. The MAB1 charge variants exhibited comparable binding activity to its native antigen, FcRn, and CD16a, except for Basic Peak 2, which showed increased binding activity towards CD16a. This finding is consistent with higher ADCC activity, where CD16a is the primary receptor involved in ADCC activity.

### 3.11. CD64 ELISA Binding Assay

CD64 (FcγRI) is another member of the Fc receptor family primarily expressed on monocytes, macrophages, activated neutrophils, and dendritic cells. The binding activity of CD64 to MAB1 and IEC-isolated peaks was also evaluated by ELISA. Despite Fc having the highest affinity with FcγRI (CD64), Basic Peak 2 did not demonstrate increased binding activity (Table 10).

## 4. Discussion

Charge heterogeneity is an important characteristic of antibodies, with C-terminal lysine from the heavy chains being one of the most common sources of this charge variation. The C-terminal lysine is encoded in both the endogenous human antibodies and the majority of therapeutic antibodies. From both in vivo and in vitro perspectives, it is generally considered not to affect antigen binding and effector functions [13,16,17,18,19,20,33]. However, Hintersteiner et al. [22] found that basic charge variants of a chimeric anti-GD2 antibody, ch14.18, demonstrated a higher affinity to the FcγRIIIa receptor when analyzed with surface plasmon resonance binding assays, although the specific composition of these basic charge variants, apart from the oxidation of M34, was not detailed. This pioneering study highlighted the need for increased attention to basic charge variants due to their potential biological functions. Subsequently, the same group of researchers analyzed several commercial therapeutic antibodies and observed that the basic peaks from Erbitux^®^, Herceptin^®^, and Xolair^®^ showed higher binding affinity to the FcγRIIIa receptor. Additionally, Erbitux^®^ and Herceptin^®^ demonstrated relatively lower EC_50_ values in an ADCC bioassay. The authors suggested that this might be due to electrostatic interactions between the basic charge variants and the FcγRIIIa receptor [23].

MAB1, an IgG_1_ therapeutic antibody in this study, shows a simple charge distribution by ion exchange chromatography with a salt linear gradient. It presents an Acidic Peak (A), a Main Peak, and two Basic Peaks (B1 and B2), representing one lysine and two lysine residues at the end of the heavy chain, respectively, as shown in Figure 1. Both isolated B1 and B2 peaks exhibited higher ADCC activity using a reporter gene biological assay. Interestingly, upon the removal of the C-terminal lysine residues in B1 and B2, the hyper-ADCC activity remained unchanged, indicating that the electrostatic interactions from C-terminal lysine may not be the cause of the higher ADCC activity.

ADCC activity was measured using an internally developed reporter gene assay designed for quality control release. This surrogate approach, based on FcγIIIa activation, offers improved repeatability, throughput, and compatibility with QC processes compared to traditional PBMC-based assays. Although this method measures luciferase activity rather than direct cell killing, results have been shown to correlate well with standard ADCC assays [27]. The assay was validated and accepted to meet regulatory requirements for product release testing. While the assay has been accepted for regulatory product release, further studies using NK cell-mediated cytotoxicity measurement may help to assess in vivo ADCC activity.

The isolated IEC peaks were comprehensively characterized for glycosylation, post-translational modifications, biophysical properties, and biological functions. All these charge variants have similar PTMs, including the deamidation level of VSNK-containing peptide, tertiary structures, DSC stability profiles, and other biological functions such as antigen binding, FcRn binding, and CD64 binding. The binding to FcγRIIIa (CD16a) was higher for peaks B1 and B2, which aligns well with ADCC results. The main difference among these charge variants was the level of MAN5 glycan, with peak B2 having the highest amount, followed by peak B1, and the Main and Acidic Peaks having the lowest activity. Consistent analytical results for the MAN5 glycan were obtained using three distinct methodologies: hydrophilic interaction chromatography, glycopeptide analysis, and MALDI mass spectrometry. The MAN5 level demonstrated a robust correlation between HILIC and peptide mapping evaluations (see Table 4 and Table 5), reflected by a coefficient of determination (r^2^) of 0.997. Comparable results concerning the significant correlation between HILIC-FLD and peptide mapping have been documented by Shipman et al. [34], who established a limit of quantitation (LOQ) ranging from 0.1 to 0.2% for both methods, thereby confirming the precision and reliability of these orthogonal analytical approaches. It is widely recognized that higher levels of afucosylated glycans, including MAN5, are associated with enhanced antibody-dependent cellular cytotoxicity (ADCC) activity [25,26,35]. The sustained higher ADCC activities observed after the removal of C-terminal lysine residues in peaks B1 and B2 indicate that these activities are attributed not to the presence of lysine residues, but rather to the presence of MAN5 glycans in a dose-dependent manner. The conclusion is summarized in Table 11, highlighting that the higher ADCC activity in B1 and B2 is not attributed to C-terminal lysine but to MAN5 glycan associated with B1 and B2 variants.

For the early work of Hintersteiner et al. [22], increased relative proportions of high mannose glycans were observed in the basic fractions, although the association between high mannose glycans and C-terminal lysine residues was not established. The association between high MAN5 glycans and C-terminal lysine residues is not clear. From a biosynthesis perspective, the C-terminal lysine variant (K2) and the presence of high-mannose glycans such as MAN5 are indicative of less processed molecular forms. Their co-occurrence reflects early-stage post-translational modifications, suggesting a connection between incomplete C-terminal processing and the early stage of glycosylation. However, this occurrence may vary on a case-by-case basis, as evidenced by different reports regarding the ADCC bioactivity functions of the basic variants.

## 5. Conclusions

While C-terminal lysine variants may not typically be classified as critical quality attributes due to their rapid in vivo clearance, their potential association with elevated ADCC activity—particularly via MAN5 glycans—warrants careful evaluation. If such an impact on bioactivity is confirmed, it may prompt regulatory scrutiny and necessitate a scientific justification regarding the control strategy for these variants to ensure therapeutic safety and efficacy.

## Figures and Tables

**Table 1:** The Average ADCC Activity of IEC Isolated Peaks.

IEC Isolated Peak	A	M	B1	B1 + CPB	B2	B2 + CPB
ADCC Activity (%)	77.40	70.08	104.87	103.76	143.00	137.04

A: Acidic Peak, *n* = 4; M: Main Peak, *n* = 9; B1, Basic Peak 1, *n* = 4; B2: Basic Peak 2, *n* = 11; B1 + CPB, Basic Peak 1 treated with CPB (Carboxypeptidase B), *n* = 4; B2 + CPB, Basic Peak 2 treated with CPB, *n* = 12.

**Table 2:** The Percent N-Linked Glycans from Unfractionated MAB1 and IEC-Isolated Peaks Analyzed by Hydrophilic Interaction Chromatography (HILIC).

Nomenclature for Glycans	Hex3HexNAc3	Hex3HexNAc3DHex1	Hex3HexNAc4	Hex3HexNAc4DHex1	Hex5HexNAc2	Hex4HexNAc3DHex1	Hex4HexNAc4DHex1	
Glycan Structure	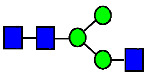	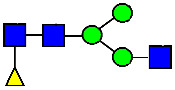	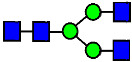	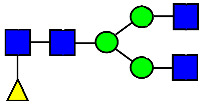	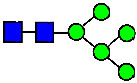	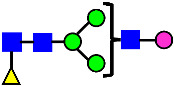	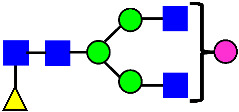	
Symbol	G0-GlcNAc	G0F-GlcNAc	G0	G0F	MAN5	G1F-GlcNAc	G1F	G1F *
Retention Time	11.299 min	12.753 min	13.363 min	14.674 min	15.891 min	16.108 min	17.393 min	17.810 min
Unfractionated MAB1	0.160%	1.452%	0.084%	22.732%	0.394%	5.057%	30.410%	10.036%
MAB1 Acidic Peak A	0.104%	1.172%	0.090%	17.262%	N/A	6.570%	26.093%	8.487%
MAB1 Main Peak M	0.104%	1.203%	0.063%	23.489%	0.205%	4.813%	32.006%	10.321%
MAB1 Basic Peak 1 B1	0.187%	2.085%	0.132%	23.872%	0.639%	6.295%	30.956%	9.797%
MAB1 Basic Peak 2 B2	0.263%	2.846%	0.193%	25.810%	2.638%	6.417%	28.511%	9.403%
Unfractionated MAB1	0.169%	1.452%	0.094%	22.839%	0.422%	5.110%	30.343%	10.095%
Nomenclature for Glycans	Hex5HexNAc3 DHex1	Hex5HexNAc4 DHex1	Hex4HexNAc3 DHex1NGNA1	Hex6HexNAc4 DHex1	Hex7HexNAc4 DHex1		Hex6HexNAc4 DHex1NGNA1	
Glycan Structure	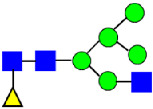	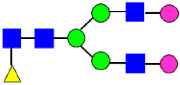	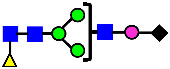	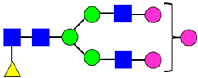	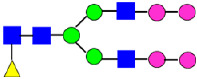		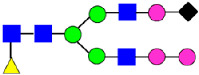	
Symbol		G2F	G1F-GlcNAcNGNA	G2FGAL1	G2FGAL2		G2FGAL1 NGNA	
Retention Time	19.274 min	20.333 min	22.036 min	22.900 min	25.379 min		27.374 min	
Unfractionated MAB1	3.315%	15.344%	1.492%	3.423%	2.090%		0.282%	
MAB1 Acidic Peak A	3.525%	15.346%	6.652%	3.939%	4.044%		1.355%	
MAB1 Main Peak M	3.672%	16.226%	N/A	3.119%	1.801%		N/A	
MAB1 Basic Peak 1 B1	4.175%	14.288%	0.240%	2.556%	1.242%		N/A	
MAB1 Basic Peak 2 B2	3.788%	12.260%	N/A	2.205%	1.055%		N/A	
Unfractionated MAB1	3.340%	14.925%	1.492%	3.372%	2.090%		0.304%	

Monosaccharide residues are N-acetylglucosamine (GlcNAc, 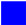), fucose (Fuc, 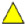), mannose (Man, 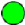), galactose (Gal, 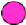), and N-glycolylneuraminic acid (NGNA, 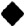). * G1F structural isomer.

**Table 3:** Glycan Structures and Theoretical Mass after 2AA-Labeling.

Nomenclature for Glycans	Glycan Symbol	Glycan Structure	Theoretical MS (Da)
Hex5HexNAc2	MAN5	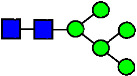	1354.479
Hex3HexNAc3DHex1	G0F-GlcNAc	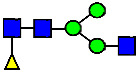	1379.511
Hex3HexNAc4	G0	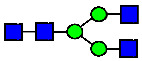	1436.532
Hex4HexNAc3DHex1	G1F-GlcNAc	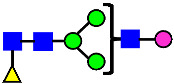	1541.563
Hex3HexNAc4DHex1	G0F	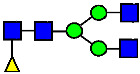	1582.590
Hex4HexNAc4	G1	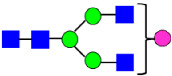	1598.585
Hex5HexNAc3DHex1		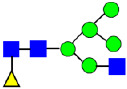	1703.616
Hex4HexNAc4DHex1	G1F	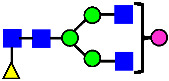	1744.643
Hex4HexNAc3DHex1NGNA1	G1F-GlcNAcNGNA	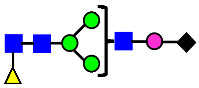	1848.654
Hex5HexNAc4DHex1	G2F	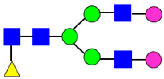	1906.696
Hex4HexNAc4DHex1NGNA1	G1FNGNA1	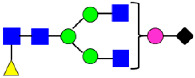	2051.734
Hex6HexNAc4DHex1	G2FGAL1	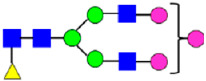	2068.749
Hex5HexNAc4DHex1NGNA1	G2FNGNA1	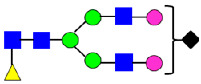	2213.786
Hex7HexNAc4DHex1	G2FGAL2	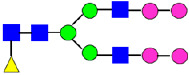	2230.802
Hex6HexNAc4DHex1NGNA1	G2FGAL1NGNA1	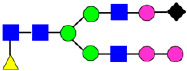	2375.839

Monosaccharide residues are N-acetylglucosamine (GlcNAc, 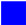), fucose (Fuc, 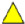), mannose (Man, 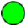), galactose (Gal, 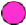), and N-glycolylneuraminic acid (NGNA, 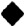).

**Table 4:** The Percentage of N-linked glycans from MAB1 and IEC-isolated peaks was measured by peptide mapping with mass spectrometry.

Nomenclature for Glycans	Hex3HexNAc3	Hex5HexNAc2	Hex3HexNAc3DHex1	Hex3HexNAc4	Hex4HexNAc3DHex1	Hex3HexNAc4 DHex1	Hex4HexNAc4	Hex5HexNAc3 DHex1	Hex4HexNAc4 DHex1
Glycan Structure	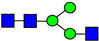	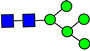	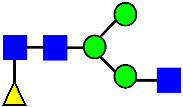	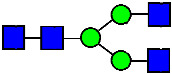	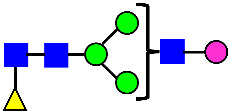	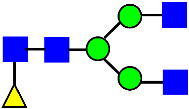	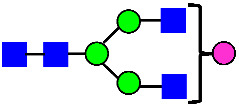	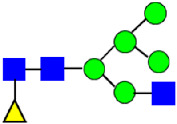	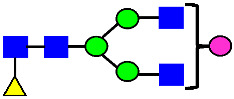
Symbol	G0-GlcNAc	Man5	G0F-GlcNAc	G0	G1F-GlcNAc	G0F	G1		G1F
Glycan Theoretical Mass	1095.3966	1216.4229	1241.4545	1298.4760	1403.5073	1444.5339	1460.5288	1565.5601	1606.5867
Unfractionated MAB1	0.227%	0.769%	3.172%	0.071%	6.221%	24.382%	0.052%	3.490%	40.623%
A1	0.192%	0.396%	3.055%	0.031%	6.514%	20.961%	0.048%	3.616%	37.389%
Main	0.174%	0.335%	3.296%	0%	5.473%	25.670%	0.045%	3.875%	42.430%
B1	0.306%	1.018%	3.786%	0.012%	7.152%	26.899%	0.066%	3.890%	40.737%
B2	0.329%	3.641%	4.936%	0.110%	7.589%	26.586%	0.144%	3.392%	38.699%
Unfractionated MAB1	0.284%	0.790%	3.512%	0%	5.935%	24.968%	0.134%	3.375%	40.534%
Glycans	Hex4HexNAc3DHex1 NGNA1	Hex5HexNAc4DHex1	Hex5HexNAc4DHex1	Hex6HexNAc4DHex1	Hex5HexNAc4DHex1 NGNA1	Hex7HexNAc4DHex1	Hex6HexNAc4DHex1 NGNA1		Hex5HexNAc4DHex1 NGNA2
Glycan Structure	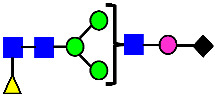	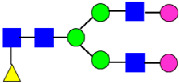	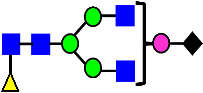	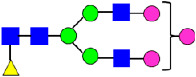	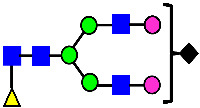	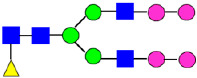	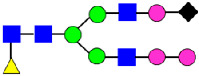		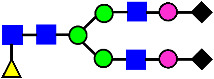
Symbol	G1F-GlcNAcNGNA	G2F	G1FNGNA	G2FGAL1	G2FNGNA1	G2FGAL2	G2FGAL1NGNA1		G2FNGNA2
Glycan Theoretical Mass	1710.5977	1768.6395	1913.677	1930.6923	2075.7298	2092.7452	2237.7827		2382.8202
Unfractionated MAB1	1.417%	14.186%	0.177%	2.541%	0.441%	1.264%	0.227%		0.060%
A1	7.151%	12.528%	0.578%	2.471%	1.731%	1.263%	1.452%		0.036%
Main	0.072%	14.568%	0%	2.197%	0.206%	1.224%	0.051%		0.108%
B1	0.158%	12.655%	0.011%	1.604%	0.172%	0.884%	0.089%		0%
B2	0.035%	11.519%	0.023%	1.523%	0.355%	0.644%	0.130%		0.027%
Unfractionated MAB1	1.312%	14.278%	0.188%	2.124%	0.438%	1.236%	0.215%		0.008%

Monosaccharide residues are N-acetylglucosamine (GlcNAc, 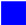), fucose (Fuc, 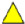), mannose (Man, 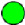), galactose (Gal, 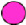), and N-glycolylneuraminic acid (NGNA, 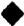).

**Table 5:** The Percent Deamidation of VSN^329^K Peptide.

Sample	VSN^329^K (%)
MAB1 Unfractionated	0.04
MAB1 Acidic Peak (A)	0.48
MAB1 Main Peak (M)	0.00
MAB1 Basic Peak 1 (B1)	0.02
MAB1 Basic Peak 2 (B2)	0.25

**Table 6:** The Emission Maximum of Unfractionated MAB1 and IEC-Isolated Peaks.

Samples	Emission Maximum (λ_max_) in nm
Reference Standard	326.68
MAB1 Unfractionated	326.76
MAB1 Pre-Acidic Peak	326.83
MAB1 Acidic Peak (A)	326.77
MAB1 Main Peak (M)	326.74
MAB1 Basic Peak 1 (B1)	326.78
MAB1 Basic Peak 2 (B2)	326.84
MAB1 Basic Peak 2 (B2) in PBS	328.43

**Table 7:** The Melting Points of the Temperature-Induced Unfolding of MAB1 and IEC-Isolated Peaks.

Samples	Tm_1_ (°C)	Tm_2_ (°C)
Reference Standard	72.17	84.67
MAB1 Unfractionated	72.79	85.29
MAB1 Pre-Acidic Peak	72.43	84.93
MAB1 Acidic Peak (A)	72.29	84.80
MAB1 Main Peak (M)	72.22	84.73
MAB1 Basic Peak 1 (B1)	71.89	85.29
MAB1 Basic Peak 2 (B2)	72.60	85.11
MAB1 Unfractionated	72.62	85.12
Reference Standard	72.48	84.87

**Table 8:** The Percentage of Biological Activities of Unfractionated Samples and IEC-Isolated Peaks Assessed by Cell-Based Proliferation Inhibition Assay.

Samples	% Biological Activity
MAB1 Unfractionated	104
MAB1 Pre-Acidic Peak	93
MAB1 Acidic Peak (A)	86
MAB1 Main Peak (M)	92
MAB1 Basic Peak 1 (B1)	88
MAB1 Basic Peak 2 (B2)	104

**Table 9:** Biacore Binding Assessment for Unfractionated MAB1 and IEC-Isolated Peaks to Native Antigen, FcRn, and CD16a.

Samples	% Binding Activity		
	Native Antigen	FcRn	CD16a
MAB1 Unfractionated	114	113	107
MAB1 Pre-Acidic Peak	87	91	108
MAB1 Acidic Peak (A)	103	107	100
MAB1 Main Peak (M)	106	108	95
MAB1 Basic Peak 1 (B1)	99	109	120
MAB1 Basic Peak 2 (B2)	96	98	193

**Table 10:** ELISA Binding Analysis for Unfractionated MAB1 and IEC-Isolated Peaks to CD64.

Samples	% Binding Activity
MAB1 Unfractionated	112
MAB1 Pre-Acidic Peak	109
MAB1 Acidic Peak (A)	99
MAB1 Main Peak (M)	92
MAB1 Basic Peak 1 (B1)	84
MAB1 Basic Peak 2 (B2)	81

**Table 11:** The Association of Elevated ADCC Activity with The Presence of MAN5 Glycan.

Sample	Acidic Peak	Main Peak	Basic Peak 1	Basic Peak 1 + CPB	Basic Peak 2	Basic Peak 2 + CPB
ADCC Activity (%)	77.40	70.08	104.87	103.76	143.00	137.04
C-Terminal Lysine	K0	K0	K1	K0	K2	K0
MAN5 Glycan (HILIC, %)	Not Detected	0.205	0.639	N/A	2.638	N/A
MAN5 Glycan (Peptide Mapping, %)	0.396	0.335	1.018	N/A	3.641	N/A

## Data Availability

All data supporting the findings of this study are available from the corresponding authors upon request.
